# Two Component Regulatory Systems and Antibiotic Resistance in Gram-Negative Pathogens

**DOI:** 10.3390/ijms20071781

**Published:** 2019-04-10

**Authors:** Anjali Y. Bhagirath, Yanqi Li, Rakesh Patidar, Katherine Yerex, Xiaoxue Ma, Ayush Kumar, Kangmin Duan

**Affiliations:** 1 Department of Oral Biology, Rady Faculty of Health Sciences, University of Manitoba, 780 Bannatyne Ave, Winnipeg, MB R3E 0J9, Canada; anjalibhagirath@gmail.com (A.Y.B.); liy34544@myumanitoba.ca (Y.L.); Katherine.Yerex@umanitoba.ca (K.Y.); Xiaoxue.Ma@umanitoba.ca (X.M.); 2 Department of Microbiology, Faculty of Sciences, University of Manitoba, Winnipeg, MB R3E 0J9, Canada; Rakesh.Patidar@umanitoba.ca; 3 Department of Medical Microbiology & Infectious Diseases, Rady Faculty of Health Sciences, University of Manitoba, 780 Bannatyne Ave, Winnipeg, MB R3E 0J9, Canada

**Keywords:** two-component regulatory proteins, antimicrobial resistance, biofilms

## Abstract

Gram-negative pathogens such as *Klebsiella pneumoniae*, *Acinetobacter baumannii*, and *Pseudomonas aeruginosa* are the leading cause of nosocomial infections throughout the world. One commonality shared among these pathogens is their ubiquitous presence, robust host-colonization and most importantly, resistance to antibiotics. A significant number of two-component systems (TCSs) exist in these pathogens, which are involved in regulation of gene expression in response to environmental signals such as antibiotic exposure. While the development of antimicrobial resistance is a complex phenomenon, it has been shown that TCSs are involved in sensing antibiotics and regulating genes associated with antibiotic resistance. In this review, we aim to interpret current knowledge about the signaling mechanisms of TCSs in these three pathogenic bacteria. We further attempt to answer questions about the role of TCSs in antimicrobial resistance. We will also briefly discuss how specific two-component systems present in *K. pneumoniae*, *A. baumannii*, and *P. aeruginosa* may serve as potential therapeutic targets.

## 1. Introduction

Antimicrobial resistance in several infectious pathogens has become a serious public health concern. As per the World Health Organization (WHO), the 21st century may well be called the post-antibiotic era [1]. The incidence of infections caused by multidrug-resistant (MDR) gram-negative bacteria is increasing worldwide [2,3]. The emergence of pan-drug-resistant (PDR) bacteria, which are resistant to all classes of available antimicrobial agents, represents a worrisome endpoint in the fight with bacterial infections [4,5]. Despite the limited reports of isolation of such resistant bacteria, there is a great concern in the medical community, as clinicians are left with very few options for treating patients with PDR bacteria.

*Pseudomonas aeruginosa*, *Acinetobacter baumannii,* and *Klebsiella pneumoniae* are well-known nosocomial pathogens; recent years have seen a worldwide rise in their multi-drug resistant and pan-drug resistant counterparts [6,7,8]. They have been included in the WHO’s list of antibiotic-resistant priority 1 (critical) pathogens [1]. They have also been annotated as being a part of the ESKAPE pathogen group [8,9,10,11] (*Enterococcus faecium*, *Staphylococcus aureus*, *K. pneumoniae*, *A. baumannii, P. aeruginosa*, and *Enterobacteriaceae*). This acronym is derived from their ability to “escape” from antimicrobial therapy. This acronym has been further modified as ESCAPE pathogens with the ‘C’ referring to *Clostridium difficile*, and ‘E’ for all *Enterobacteriaceae,* including *E. coli*, *Proteus* spp., and *Enterobacter* spp. [12]. Studies have documented increasing resistance rates in *P. aeruginosa* clinical isolates to fluoroquinolones, cephalosporins, and carbapenems [13]. *A. baumannii* and *K. pneumoniae* are now being recognized as emerging pathogens in many medical facilities [14,15]. According to the National Nosocomial Infections Surveillance (NNIS) data, the proportion of infections due to *Acinetobacter* spp. has been steadily increasing, and now accounts for ∽7% of intensive care unit (ICU)-related pneumonias [16]. Infections due to multidrug-resistant *A. baumannii* have been associated with increased lengths of hospital and ICU stays [14]. *K. pneumoniae* is also a well-recognized nosocomial pathogen, and an important cause of pneumonia and urinary tract infections in ICU settings [15]. Since the early 1990s, many reports of extended-spectrum β-lactamase (ESBL)-possessing *K. pneumoniae* have emerged [15]. In surveillance studies, resistance to third-generation cephalosporins amongst *K. pneumoniae* has reached ∼15–20%, and ciprofloxacin resistance has ranged from ∼10–50% [17,18]. Recently, outbreaks of carbapenemase-producing *K. pneumoniae* have been reported, threatening the use of this class of antimicrobial agents [17,18].

Even though resistance mechanisms in these organisms have been increasingly explored, limited information is available regarding the role of their sensory mechanisms in resistance. Sensing is the first step for bacterial defense against extrinsic environmental stressors such as antibiotic exposure and potentially plays a role in the evolution of resistance mechanisms. Two-component regulatory systems (TCSs) in bacteria act as key sensory pathways that enable microbes’ adaptation in both the environment as well as the host [19]. Studies have suggested that TCSs may play an important role in the survival and development of antimicrobial resistance [20]. This review aims to explore the connection between TCSs and antimicrobial resistance in pathogenic gram-negative bacteria. We will focus on three key bacteria: *P. aeruginosa, K. pneumonia*, and *A. baumannii*, which pose serious threats to human health [21,22,23]. We believe these organisms also share a commonality in terms of the mechanism of antibiotic resistance.

## 2. Antimicrobial Resistance in Gram-Negative Pathogenic Bacteria

The development and emergence of antimicrobial resistance is a complicated process and yet occur soon after the introduction of novel anti-microbial drugs. Resistance to antibiotics can develop either by spontaneous mutations [24] or by the acquisition of resistant genes [25]. The origin of antibiotic-resistant genes may be present on commensal [26] or environmental bacteria [27]. To fully understand the acquisition and spread of antibiotic resistance among the human bacterial pathogens, these ecosystems should be taken into consideration.

Development of resistance is thought to be an ongoing evolutionary process. A genetic change in the form of mutation often occurs naturally. Such mutations can influence the ability of a cell to grow and survive in the presence of environmental stressors such as antimicrobials [28]. The selection of mutant is dependent on the intensity of selective pressure, the immune status of the host, the size of the pathogen population, the presence of other microorganisms and lastly the geographical topography [28,29]. Often, random mutations of the genes encoding for the antibiotic lytic enzymes give rise to modified catalysts with increasingly extended spectra of resistance [30]. One example is the β-lactamase encoding gene. The β-lactamase encoding genes are ancient and have been isolated in strains from remote environments [31,32]. The plasmid-encoded β-lactamase, TEM, has been shown to be related to a variety of enzyme families, providing hints to its adaptability [33].

**Figure 1:**
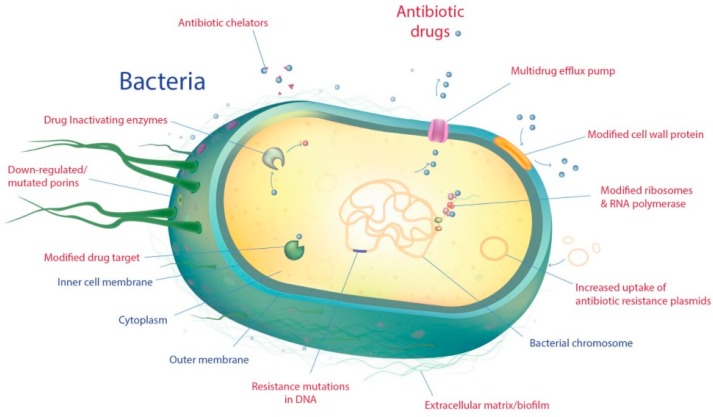
Key resistance mechanisms in gram-negative pathogenic bacteria.

Antimicrobial resistance can develop through several mechanisms. These can be broadly categorized into two main types; intrinsic and acquired. Intrinsic resistance mechanisms include inherent bacterial defenses such as altered outer membrane permeability and hyperactive efflux pumps; whereas, the acquired resistance mechanisms involve horizontal gene transfer and acquisition of genetic elements. Here we will discuss these mechanisms with respect to *P. aeruginosa*, *K. pneumonia*, and *A. baumannii*. Figure 1 highlights the key resistance mechanisms in pathogenic bacteria.

### 2.1. Intrinsic Resistance

Intrinsic gene functions leading to naturally resistant phenotypes have given rise to the term ‘resistome’ [34]. Resistome of a microorganism includes all the genes and their products that contribute to its antibiotic resistance [35]. Understanding the resistome is critical as it forms the basis for horizontal gene transfer and the eventual emergence of antimicrobial resistance [35,36]. The search of *P. aeruginosa* PAO1 genome, for example, reveals several genes encoding enzymes for resistance to chloramphenicol, aminoglycoside, and β-lactam antibiotics [37]. Resistome analysis in multidrug-resistant isolates of *K. pneumoniae* revealed an average of 11–13 acquired resistance genes along with the extended-spectrum beta-lactamase genes and the AraC-type regulator, which confers resistance to virtually all antimicrobial agents available in clinical practice [36,38,39]. The genome of *A. baumannii* encodes a variety of different β-lactamases, including metallo-enzymes that confers resistance to carbapenems, as well as resistance-nodulation-cell division (RND)-type multidrug efflux pumps (AdeABC, AdeFGH, and AdeIJK) [40,41,42,43].

The outer membrane (OM) in gram-negative bacteria plays a major role in pathogen-host interaction and forms a selective permeability barrier. OMs, like other biological membranes, are fundamentally built as a bilayer of lipids. As such, lipid bilayers permit little permeability for hydrophilic solutes, including most nutrients and many antibiotics [44]. They contain protein-formed channels, allowing the influx of nutrients and the extrusion of waste products [44]. Porins are one such class of constitutively expressed nonspecific or substrate specific diffusion channel-forming proteins [45]. The properties of these porins are significant for the intrinsic level of antibiotic resistance in gram-negative bacteria. The major outer membrane porin of *P. aeruginosa* (OprF) transports solutes at least two times slower compared with that of bacteria, such as *E. coli* [46]. A recent study in *K. pneumoniae* suggested that porin deficiency is a widespread phenomenon in MDR resistant isolates [47,48,49]. The genome of *K. pneumoniae* encodes for several key porins, however, OmpK35, OmpK36, and OmpK37 are most widely associated with AMR. Most susceptible clinical isolates of *K. pneumoniae* express both OmpK35 and OmpK36 porins, while most extended spectrum β-lactamases encoding *K. pneumoniae* express only the OmpK36 or none [50,51]. Very often loss of one type of porin is compensated by expression of others, adding to the already complex role of porins in antimicrobial resistance. The major *A. baumannii* porin, OmpA, has been shown to play a crucial role in AMR [52]. OmpA facilitates AMR by extrusion of antibiotics from the periplasmic space through the outer membrane and by interacting with inner membrane efflux systems [52] facilitating surface motility [53] and biofilm formation [54].

Bacterial efflux pumps represent another important mechanism for limiting antibacterial molecules inside the cell or from their targets. Efflux pumps are central to the adaptation and survival of the cell in various environments. These are divided into families such as the RND; major facilitator superfamily (MFS); small multidrug resistance (SMR), and multidrug and toxin extrusion (MATE) family of proteins. Another class of proteins, known as the ATP binding cassette (ABC) transporters, have been found to be present in various pathogenic bacteria and have been shown to be involved in antibiotic resistance. Contribution of efflux systems to clinically important antibiotic resistance has been described in *P. aeruginosa* (MexAB-OprM, MexCD-OprJ, MexEF-OprN, and MexXY-OprM) [55,56,57,58], *A. baumannii* (AdeABC, AdeFGH, CraA, AmvA, AbeM, and AbeS) [40,43,59,60,61], and *K. pneumoniae* (TolC, AcrAB, KocC, and KexD) [62,63,64]. Readers are referred to excellent reviews by Yoon et al. [61] Pulzova et al. [49], and Zowalaty et al. [65].

### 2.2. Acquired Resistance

Modifying enzymes catalyze reactions including acetylation, phosphorylation, and adenylation. The steric hindrance caused decreases the affinity of the drug for its target, resulting in AMR [66,67]. One example is aminoglycoside acetyltransferase (AAC) found in *Pseudomonas* and *Acinetobacter* which can inactivate most aminoglycosides including amikacin and gentamicin [68,69]. These enzymes are either acquired through horizontal transfer of plasmids or transposons or through spontaneous mutations which expand the functionality of encoding genes [70]. The other mechanisms are target modification and lipopolysaccharides (LPSs) modification [71]. Where antibiotics affect bacterial cell-wall or target cell division, target modification works by either acquisition of binding proteins that do not affect cell-wall or acquisition of mutations within the RNA polymerase, DNA gyrase or topoisomerase IV, modification of ribosomal proteins, or protection of the target site by another protein altogether [17,67,72].

The other more complex mechanism for evading external stressors such as antimicrobials is the formation of structured communities known as biofilms [73]. Biofilms are comprised of an exopolysaccharide matrix surrounding the bacterial communities, with well-established channels for nutrients and water inflow as well as waste outflow [73]. The exopolysaccharide matrix limits the penetration of antibiotics while the proximity allows for horizontal gene transfer from the persister cells [74,75]. Persister cells are those subgroups that have survived an antimicrobial exposure and can give rise to resistant colonies [75]. Further, cells inside the biofilms grow relatively slowly and present low metabolic activity which is detrimental to the activity of most currently available antibiotics [75]. Interestingly, sub-inhibitory concentrations of aminoglycosides, especially tobramycin, have been shown to induce biofilm formation in *P. aeruginosa* [76]. Azithromycin has been shown to inhibit the expression of the small RNAs *rsmY* and *rsmZ*, a process that depends on the GacA/Rsm signal transduction pathway. GacA/Rsm pathway is known to positively control quorum sensing and reciprocally control biofilm formation in *P. aeruginosa* [77]. Readers are referred to reviews by Høiby et al. 2010 [70], Philip S. Stewart, 2002 [74], and Ahmed et al. 2018 [78].

Comparative genomic analysis suggests that horizontal gene transfer (HGT) plays a significant role in determining the genetic repertoire of the clinical isolates of pathogenic bacteria [25,79,80]. Genomic diversity is, in part, attributable to the acquisition of genetic material that has integrated into the chromosome at a relatively limited number of sites [72]. Acquired mobile genetic elements (plasmids, insertion sequences, transposons) mobilize the antimicrobial resistance genes and can confer resistance to the major classes of antimicrobials among different bacteria. Resistance to environmental stressors is triggered by contact between bacterial sensing systems and the immediate extrinsic environment. The interaction between the sensing systems within the bacteria and the external environment leads to adaptive physiological changes through modulation of gene expression. Two-component regulatory systems detect physical and chemical changes in the environment and then relay this signal to the cytoplasm, where the modulation of gene expression occurs.

## 3. Two-Component Regulatory Systems in Gram-Negative Pathogenic Bacteria

TCSs have been known to regulate a wide variety of cellular functions in response to environmental signals such as nutrient limitation, oxygen availability, phosphate limitation or osmolarity, and antimicrobial agents [81,82,83]. For instance, in *P. aeruginosa* PilG/PilH mediates pili production under yet unknown signals and NarX/NarL is involved in nitrate sensing and respiration, biofilm formation and motility [84]. AlgZ/AlgR mediates alginate production under osmolarity and nitrate signals in mucoid strains [85]. PhoR/PhoB senses inorganic phosphate and is involved in regulating quorum sensing and swarming motility [86]. PfeS/PfeR senses enterobactin mediated iron acquisition [87] while FleS/FleR mediates adhesion and sense mucins [88]. GacS/GacA controls virulence in response to unknown signals and CbrA/CbrB senses various carbon sources and modulates metabolism, virulence and antibiotic resistance [89]. CheA/CheY regulates chemotaxis in response to magnesium [82,83,90].

**Figure 2:**
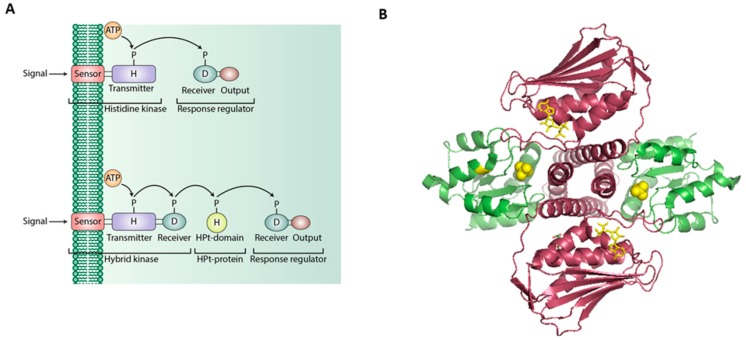
Schematic diagram of the functions and domains of sensor kinase and response regulator proteins in TCSs and HHK-mediated phosphorelays. (**A**) Representation of the classical TCS and phosphorelay signaling systems. (**B**) Structure of the complex between the entire cytoplasmic portion of *Thermotoga maritima* class I histidine kinase (magenta) and its cognate, response regulator (green) (PDB entry code 3 DGE) [91].

Typically, a TCS consists of a sensor kinase with a conserved histidine residue [H box] also known as histidine kinase [HK] which senses external signals and transfers a phosphate molecule to the response regulator [RR] with a conserved aspartate residue which then mediates cellular response towards the external stimuli (Figure 2). In contrast to the orthodox TCS systems, the phosphorelay system mediated by hybrid histidine kinase (HHK) is also shown.

Bacterial HKs are classified into five types (Type I, II, III, IV, and CheA). Among the five HK family types, Type I and II are found to be genetically related, but type III and IV are not [92]. The likeness between Type I and II is based on the presence of orthodox kinase domains, which contain the N, G1, F, and G2 consensus motifs. Type III and IV HKs possess so-called unorthodox kinase domains in which N1 of the N-box motif is either a glycine (Type III) or a proline (Type IV) residue, the F box is absent, and the G2 motif is truncated. Within the Type I group, three separate subtypes exist: The Type IA group contains 12 HKs, the Type IB group contains the hybrid HKs and the Type IC group contains three HKs, including the nitrogen regulator *ntrB*. The kinase domain of CheA is characterized by insertions between the N and G1 boxes and the G1 and F boxes. The N-box of CheA contains a histidine residue at the N1 position [92]. Secondary structure analysis of HKs predicts a helix–loop–helix structure important for signal recognition [92].

**Figure 3:**
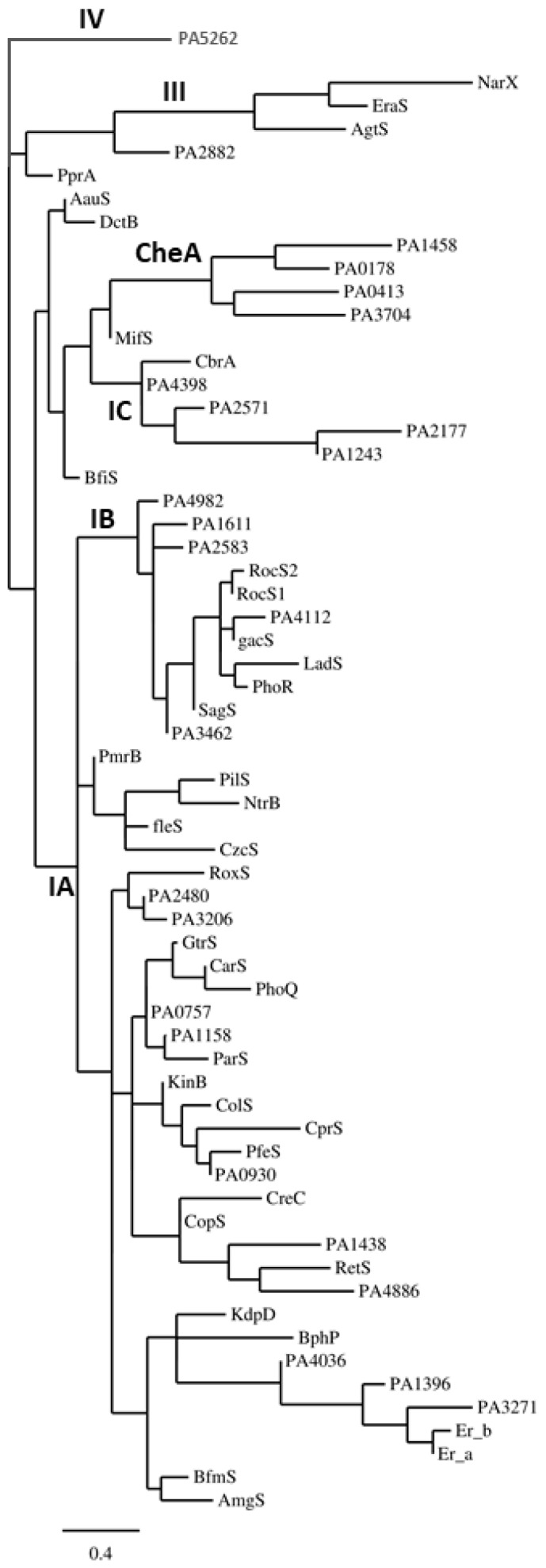
Phylogenetic analysis of the HKs of *P. aeruginosa.* Known HKs were aligned followed by phylogenetic analysis at *Phylogeny.fr* [93,94].

The HK types found in *P. aeruginosa* genome were assembled in Figure 3 using the gene tables of the completed genomes listed in the *Pseudomonas* genome database (http://www.pseudomonas.com). This was compared with the available classification on MiST3 (mistdb.com). The protein sequences were aligned in *Phylogeny.fr* using MUSCLE for multiple alignments, PhyML for tree building, and TreeDyn for tree rendering [93,94]. Of the total HKs identified in *P. aeruginosa*, one cluster within the Type IA group (PA1396, 1976, 1992, 3271, and 4936) contains orthodox kinase domains while the H-box motifs containing a non-polar residue at position 4 and a glutamine residue at position 5. This clade of HKs forms a distinct branch within the Type IA group. In addition, a cluster of HKs in the Type IC group possesses the consensus H-box motif HDLNQPL in which the asparagine residue replaces the typical positively charged residue at position 4, but the glutamine residue at position 5 is highly conserved. *P. aeruginosa* lacks Type II HKs and possesses four CheAs [90]. Two HKs, PA3078, and PA4380, which cannot be assigned to the defined type, are categorized as unclassified. Helix–loop–helix structures are also predicted in the H-box region of the Type III HKs. The H to N distances for each of the HK types are similar to those found in the different HK types of *E. coli*. Finally, the majority of the HKs are found in operons with cognate RRs [92].

**Figure 4:**
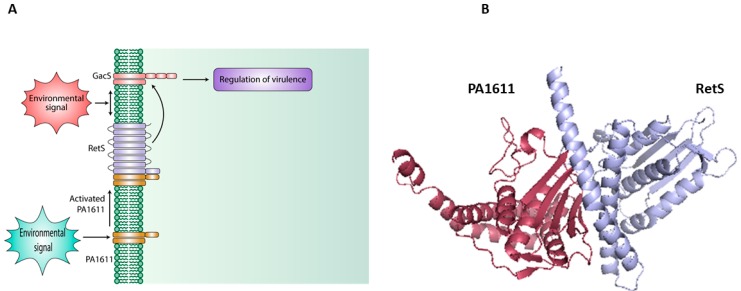
Schematic diagram of the direct protein-protein interaction mediated signaling using the PA1611-RetS interaction model as an example [96]. (**A**) Canonical TC Sensor GacS phosphorylates its response regulator GacA to regulate virulence in *P. aeruginosa*; however, under yet unknown environmental signals, HHK PA1611 is activated and binds to HHK RetS. Under such conditions, GacS is again free to phosphorylate its cognate response regulator and mediate downstream signaling. (**B**) A docked complex for homology models for PA1611 and RetS showing predicted interacting surfaces.

Apart from the classical HK and RR domains, a histidine phosphotransfer protein (Hpt) may be encoded in the same operon. These Hpt proteins are sometimes present either as isolates, as seen in orphan histidine kinase, or along with HHK for phosphotransfer [95]. The division of domains in phosphorelays provides additional checkpoints for phosphorylation and may serve to integrate signals for a collective response as cross-talks are allowed in such systems [95]. Recent studies also suggest a non-phosphotransfer based signaling pathway (Figure 4), in which two HKs can interact directly to elicit a certain response by controlling downstream responses of the non-cognate binding partners [96].

### 3.1. One-Component Signaling Systems

One-component systems (OCSs) can be defined as proteins that contain both input and output domains but lack typical histidine kinase and response regulator domains of TCSs. OCSs represent the simplest model for signal transduction by a single protein. One example is RocR in which PAS and HTH are input and output domains respectively. As reported by Ulrich et al., one-component systems were shown to numerically dominate over TCSs in bacteria which was also co-relatable to their genome size [97]. One component signal transduction proteins also demonstrate less conservation within their input and output domain architecture than TCSs [97]. Based on domain architecture, one component systems seem to be the precursors for two-component signaling systems. The structure and abundance of TCSs also suggest that the addition of a histidine kinase might have been an evolutionary step at intercepting external signals [98].

*P. aeruginosa* PAO1 (genome size 6.2Mbp) encodes for 435 OCSs (mistdb.com) and 130 TCSs (41 HK; 17 HHK; 69 RR; others 5). This is not very different from clinical strains *P. aeruginosa* 138,244 (widely disseminated and associated with multidrug resistance) (428 OCSs and 134 TCSs), *P. aeruginosa* 152,504 (rare allele) (466 OCSs, and 145TCSs). However, in contrast, *K. pneumonia* subsp. *pneumoniae* strain HS11286 (genome 5.3Mbp) encodes for 387 OCSs and 66 TCSs (27 HK; 5 HHK; 32 RR; Others 2), and *A. baumannii* (genome 4.3 Mbp) exhibits 235 OCSs and 29 TCSs (11 HK; 3 HHK; 15 RR).In contrast to the one-component systems, the counterparts of two-component signaling systems are highly conserved, and the RRs are more conserved than HKs.

### 3.2. Hybrid Histidine Kinase [HHK] and Direct-Interaction-Mediated Signaling

Hybrid histidine kinase proteins have, in recent years, become an emerging group of proteins with demonstrated roles in complex signaling mechanisms [96,99,100,101]. *P. aeruginosa* PAO1 encodes for 15 HHKs while its environmental counterpart *P. aeruginosa UCBPP-PA14* has 18. *K. pneumoniae* subsp. *pneumoniae HS11286* encodes for 5 and *A. baumannii* encodes for 3 HHKs in their respective genomes. HHKs in *P. aeruginosa* PAO1 includes those involved in a key regulatory pathway regulating biofilm formation and virulence (RetS; GacS; LadS; PA1611) [13,96,100,102]. HHKs in *K. pneumoniae* include the ArcB (oxygen sensor) [103]; EvgS (capsular polysaccharide) [104]; BarA (carbon metabolism) [105]; RcsC (motility and capsular synthesis) [39]; however, those in *A. baumanii* (IX87_RS17040, IX87_RS13225, IX87_RS03185) remain largely unexamined. Interestingly, HHKs have been shown to function via both canonical phosphotransfer-mediated signaling as well as by direct protein–protein interaction-mediated signaling one example is the LadS-GacS-RetS-PA1611 system in *P. aeruginosa* [96,100,101]. Previously, this mechanism has been observed in the PmrB/PmrA TCS in *Salmonella* [106]. The PmrB/A TCS is required for resistance to acidic environment and antibiotic stresses [106]. PmrD, another regulatory protein from the same operon, binds to and protects the phosphorylated form of PmrA from the phosphatase activity of its cognate sensor, PmrB [106].

In the phosphorelay systems involving HHKs, the Hpt protein serves as the phosphodonor to the terminal response regulator which eventually mediates a cellular response via the output domain. The Hpt protein is also capable of receiving phosphor from HHKs and functioning as an independent protein [107]. An Hpt may serve as a point of signal integration or transmission of signals between two non-cognate TCSs. Although as much as 90% of the TCSs in eukaryotes use hybrid HKs, only 20% of the characterized prokaryote genomes encode hybrid kinases whereas in archaea the number is only 1% [108]. This is explained by the fact that the larger size of a eukaryotic cell necessitates complex signaling and multi-step phosphorelays. With the modular organization in the phosphorelays, phosphorylation at any level may lead to activation of the output domain [109].

Recent studies have highlighted interactions between the HHKs and other auxiliary proteins such as those involved in biofilm formation and regulating efflux pumps in pathogenic bacteria. One classic example is the SagS HHK in *P. aeruginosa* [110,111]. SagS is expressed during the biofilm development stages and regulates c-di-GMP levels as well as activates MexAB-oprM and MexEF-oprN systems [112,113].

As signaling pathways must act in a combinatorial fashion for normal cellular functioning, there is a possibility of inter-signaling system- information transfer. The direct interaction between TCS proteins is a recently uncovered strategy that the bacteria use to integrate signals other than those detected by a given sensor [114]. These proteins may function as negative or positive regulators of the TCSs involved. Regulatory proteins may also target the response regulators to either protect from dephosphorylation or cause dephosphorylation [115]. Apart from pure phosphotransfer-based signaling, some sensor kinases may also function by either de-phosphorylating the response regulator or protecting the RR from de-phosphorylation. They can perform these functions by binding or sequestering their cognate/non-cognate partners.

Interestingly, more and more reports have emerged suggesting that TCS–TCS direct interaction may also affect signaling states and act as signal transducers for interacting proteins [116,117,118,119,120]. A clear correlation between structural properties, domain interaction, and signaling states is suggested. Though a single TCS may function independently in several different ways, the interaction between TCSs can be described with Boolean operators with the analogy to the neural networks as was first noted in *E. coli* [121,122]. In *E. coli*, ArcB, TorS, RcS, and EvgA have been shown to signal through RRs in non-cognate clusters. These recruitment mechanisms are crucial for chemotaxis and sporulation systems [98,123]. In such case, HHKs are a particularly interesting group, which are able to function alone or in conjunction with diverse cognate and non-cognate partners to form signaling complexes [86,124,125,126,127,128]. A multilayer regulation provides an organism greater control over environmental responses. For cells to function as one single unit, the signaling pathways must act in a combinatorial fashion. Thus, apart from sensing the external signal, there is a possibility of inter-signaling system-information transfer, with HHKs being involved in forming signaling complexes [86,124,125] and play a role in multidrug resistance [100,126,127,129].

## 4. Role of Two-Component Regulatory Systems in Antimicrobial Resistance in Gram- Negative Pathogenic Bacteria

The current knowledge on bacterial genome sequences has made it possible to investigate, identify, and predict two-component regulatory proteins as well as their interacting partners. Two-component regulatory proteins in *P. aeruginosa* have been widely studied and reviewed. However, the same is not the case for *K. pneumoniae* and *A. baumannii*. Table 1 provides a comprehensive listing of available knowledge about various two-component regulatory systems in these three pathogens.

**Figure 5:**
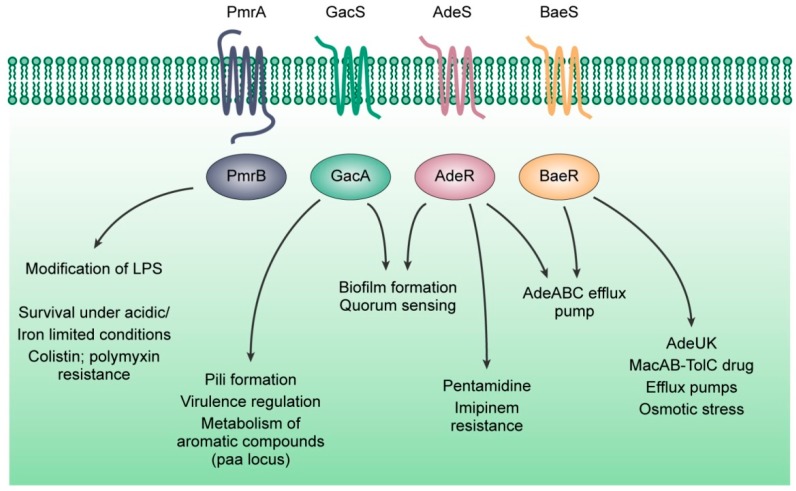
The known roles of PmrAB, GacSA, AdeRS, and BaeSR, two-component regulatory systems in antimicrobial resistance.

We analyzed two-component regulatory proteins between these three pathogens and found a surprising conservation in the PmrAB, GacSA, AdeRS, and BaeSR, systems. Among the HKs and RRs, we observed a greater degree of conservation in the RRs. We will discuss the available pool of knowledge on these four systems and their role in antimicrobial resistance (Figure 5). We focus only on these four TCSs [217,218] as they exist across the three bacteria under discussion and are related to antimicrobial resistance.

### 4.1. The PmrAB System

PmrAB TCS was shown to be involved in the LPS modification in *P. aeruginosa*. The genes *pmrB* and *pmrA* encode for the sensor histidine kinase (PmrB) and its cognate response regulator (PmrA), which, once phosphorylated, activates the *pmrC* and *pmrHFIJKLM* as well as downstream genes. Genetic targets of PmrAB in *P. aeruginosa* include the *cprA* gene required for polymyxin resistance [20] and the *pmr* genes (*PA3552–PA3559*) for resistance from antimicrobial peptides [230]. The HK PmrB in *P. aeruginosa*, *K. pneumonia,* and *A. baumanii* showed a BLOSUM similarity score of 29%. PmrA in *P. aeruginosa*, *K. pneumonia,* and *A. baumanii* showed a 43% similarity (BLOSUM). Phylogenetically, PmrA in *P. aeruginosa* is more closely related to *K. pneumoniae* as compared to that of *A. baumanii*. Interestingly, a closer look at the past studies reveals a far more functional similarity.

Mutations in the *pmrAB* operon (polymyxin resistance) have been linked to enhanced resistance to antimicrobial peptides as well as survival in chronic infections. The PmrAB system (PA4776–PA4777) was first identified in *P. aeruginosa* clinical isolates with resistance to polymyxins. It was observed that a mutation in the PmrAB locus resulted in resistance to polymyxins and other cationic antimicrobial peptides (CAPs) [134,231,232]. Further PmrAB was shown to have a role in survival and persistence in chronic lung infections. A mutation in the PmrB resulted in an increased ability to survive in a mouse model of chronic respiratory infection as compared to both the wild type as well as those adapted to the mouse lung but lacking the mutation [233]. Recent studies have argued that PmrB mutants were, in fact, more susceptible to antimicrobials, such as ciprofloxacin, colistin, gentamycin, polymyxin B, tobramycin, and tetracycline [234]. The same study also identified at least 216 proteins that were differentially regulated in a PmrB mutant. Interestingly, the PmrB mutant was found to show enhanced resistance to host-derived antimicrobial peptides [234].

To survive environmental stressors, one of the unique mechanisms that the pathogenic bacteria employ is the ability to remodel their outer membranes. This remodeling occurs, mainly at the level of lipid A in the lipopolysaccharide (LPS). The remodeling of lipid A occurs in a PhoPQ-PmrAB dependent manner by palmitoylation or deacylation, or both, by the addition of 4-aminoarabinose (L-Ara4N) or phosphoethanolamine (pEtN) [235]. The addition of L-Ara4N is considered the most effective by decreasing the net negative charge of the membrane to zero [46]. Thus, this would reduce its binding to polymyxins, resulting in resistance. The second, PEtN modification, decreases the net charge from −1.5 to −1 [46].

Similar to that in *P. aeruginosa, pmrAB* TCS in *K. pneumoniae* has been shown to regulate lipopolysaccharide modification [236]. In *A. baumanii,* mutations in PmrAB have been shown to be associated with colistin resistance [237], however, the mechanism is still unclear. It has been suggested that mutations in PmrA or B, or both, could result in lipid modifications [238]. The HK gene *pmrB* seems to be the more common site for bacterial mutations compared to the RR gene *pmrA*. Interestingly, the acquisition of colistin resistance was also found to be associated with decreased virulence and fitness [239]. In contrast, recent studies on PmrB mutant in *A. baumanii* have shown no reduction in virulence or fitness [240]. It has been suggested that PmrB senses acidic pH (pH 5.5), low magnesium levels and iron limitation, and also increases the survival under antimicrobial stress [241,242,243] via yet unknown mechanisms.

### 4.2. The GacSA System

The GacSA TCS is one of the most widely studied systems in *P. aeruginosa*. GacS is an HK and GacA is the RR. Phosphorylation of GacS is under the control of hybrid sensor kinases, RetS (PA4856) [99], PA1611 [129], and LadS (PA3974) [101]. These three HHKs are known to bind to GacS under yet unknown environmental stimuli to reciprocally control the acute-chronic disease transition in *P. aeruginosa*. Once phosphorylated, GacA activates the transcription of two small regulatory RNAs, RsmZ (PA3621.1), and RsmY (PA0527.1) [99]. RsmY/Z control the activation of the RNA-binding protein RsmA (PA0905) [244]. RsmA is known to regulate genes of the Type III secretion system, type IV pili formation and iron homeostasis while repressing QS, Type VI secretion and potentially other transcription factors [245]. The MexEF-OprN pump in *P. aeruginosa* has been found to remove several antibiotics, *Pseudomonas* quinolone signal and specific quorum sensing molecules from the cell [246]. RsmA has been shown to control the expression of the MexEF-OprN pump [247]. A *rsmA* mutant demonstrated results in activated expression of the genes encoding the MexEF-OprN pump [247]. The GacSA system is also involved in antibiotic resistance, through RsmA/RsmZ, to three different families of antibiotics: tobramycin, ciprofloxacin, and tetracycline [248]. Further, biofilms known to be resistant to available antibiotics in *P. aeruginosa* are affected primarily by the *pel* and *psl* operons and broadly by the modulation in intracellular c-di-GMP levels [249]. c-di-GMP is a second messenger shown to promote biofilm formation and antimicrobial resistance in *P. aeruginosa* [249]. Both the *pel* and *psl* operons are post-transcriptionally regulated by the RetS-LadS systems via RsmY/Z [99,100,250]. c-di-GMP exerts a broader control via its effect on a variety of regulatory proteins and RNAs. Previously, it has been shown that c-di-GMP levels, are modulated by the diguanylate cyclase WspR (PA3702), which is involved in the switch between acute and chronic infection phase and is shown to be dependent on RsmY/Z [251]. Small colony variants (SCV) of P. *aeruginosa* clinical isolates are known to exhibit hyper biofilm formation, hyper pilation and demonstrate enhanced resistance to several antibiotics [252]. Often the SCV phenotype is associated with elevated intracellular levels of c-di-GMP [142]. Studies have also shown that a mutation in the PmrAB system is associated with SCVs and hyperbiofilm phenotypes.

The *amgRS* operon encodes a membrane stress-responsive TCS found to be linked to intrinsic aminoglycoside resistance in *P. aeruginosa*. The AmgS is the sensor kinase and the AmgR is the response regulator. AmgRS TCS has been shown to provide resistance against aminoglycoside-related membrane damage [145]. AmgRS was shown to be activated in the presence of aminoglycosides which in turn promoted the *mexXY* expression [145]. It was observed that while overexpression of the AmgRS system slightly reduced the colony size of wild-type PA14, the SCV formation was enhanced significantly when GacA was overexpressed simultaneously, [232].

Similar to *P. aeruginosa,* the GacSA system in *A. baumannii* was shown to be a global regulator of virulence, pili, biofilm formation and resistance to host-derived antimicrobial peptides and motility [222]. It was also shown that GacSA played a key role in attachment to abiotic surfaces, arginine metabolism, and biofilm formation [253]. A GasS mutant of *A. baumannii* showed decreased virulence towards *Candida albicans* [254] and was unable to use citrate as the carbon source [255]. The phenylacetic acid (PAA) pathway is crucial to the metabolism of aromatic compounds and environmental pollutants in bacteria. In *A. baumanii*, the PAA pathway is encoded by the *paa* operon and it was observed that the deletion of *gacS* resulted in repression of the entire *paa* operon [222].

A search for the GacSA system in *K. pneumoniae* identified BarA with 93% similarity to the GacS HK and UvrY with 100% similarity to the GacA RR in *P. aeruginosa*. BarA and UvrY correspond to KpST66_3517 and KpST66_0986 in *K. pneumoniae* genome [256]. These proteins consist of an N-terminal cytosolic domain, a canonical pair of transmembrane regions linked by a periplasmic bridge, a transmitter domain containing a conserved histidine residue, a central receiver domain with a conserved aspartate residue, and a C-terminal phosphotransfer domain with a conserved histidine residue [257]. To date, there have been few studies on this TCS and its role in antimicrobial resistance in *K. pneumoniae*.

The carbon storage regulation (Csr) system has been shown to have a major impact on regulation of carbon metabolism pathways, motility, and biofilm formation [256]. The Csr system is composed of small regulatory RNAs possessing repeated sequence elements that allow them to interact with multiple copies of the RNA binding proteins, thereby preventing its regulatory interaction with its mRNA targets downstream [256]. UvrY has been shown to activate the expression of the noncoding *csrB* and *csrC* RNAs in *E. coli* [257]. This, in turn, sequesters CsrA and prevents it from activating downstream genes. CsrB is a carbon source utilization system which has been shown to integrate signals from the UvrY-BarA TCS [105]. Functional studies for the UvrY-BarA in *Escherichia coli* have shown roles in catalase expression [258,259], biofilm formation [256] and quorum sensing [260].

Unlike PmrAB, AdeRS, and BaeSR, GacSA is not a contiguous operon in any of the three pathogens discussed above suggesting that GacSA may be involved in crosstalk between different TCSs. The full extent of its role in these pathogens yet remains to be understood.

### 4.3. The AdeRS and the BaeSR Systems

The AdeRS two-component regulatory system is composed of the AdeS as a sensor kinase, whereas AdeR is the RR. The AdeRS system is one of the best characterized TCS in *A. baumanii.* The AdeRS TCS has been shown to be involved in a more global regulation of gene expression in *A. baumanii* either directly in sensing cell density/growth, or indirectly as in sensing osmolality via the BaeSR system [261].

Efflux pumps are usually regulated by regulatory proteins adjacent to them. There are few reports of efflux pumps regulated by TCSs including the NorA pump in *S. aureus* [262]. Interestingly, AdeRS has been demonstrated to control the expression of *adeABC* efflux pump in *A. baumanii* [59]. AdeABC efflux pump, a three-component system, and a member of the RND family has been shown to play a role in resistance to aminoglycosides, tetracycline, erythromycin, chloramphenicol, trimethoprim, fluoroquinolones, and tigecycline [263]. AdeABC consists of AdeA, the inner membrane fusion protein, AdeB the transmembrane component, and AdeC the outer membrane protein. *A. baumanii* ATCC 17978 has two *adeA* genes and one *adeB* gene, but lacks the *adeC* [264]. The overexpression of AdeABC is also associated with increased virulence, which probably explains why *adeRS* mutations are frequently observed in clinical isolates [61,216,265,266]. Recent studies showed that AdeRS, directly or indirectly, regulates 579 genes, most notably those involved in the expression of efflux pumps, biofilm formation and virulence in a *Galleria mellonella* larvae infection model [267]. Intriguingly, some outcomes of the AdeRS deletion appeared to be strain specific. Further truncations or point mutations within AdeR or S leads to activation of AdeABC efflux system and results in multidrug resistance [268,269].

A search for AdeR homolog in *P. aeruginosa* resulted in a yet unknown response regulator belonging to OmpR family, containing a DNA-binding response regulator with 91% similarity (WP_033958295.1) and 93% similarity in *K. pneumonia* (WP_004199992.1). The roles of either are not known yet.

The BaeSR TCS was first discovered in *E. coli* [270] and *Salmonella enterica* serovar *Typhimurium* [217]. The BaeSR TCS consists of an inner-membrane-bound BaeS HK, and a cytoplasmic RR, BaeR. Genome analysis of *A. baumannii* ATCC 17978 shows that the coding sequences of *baeR* (A1S_2883) and *baeS* (A1S_2884) are arranged sequentially, suggesting that the two genes may be co-transcribed as one operon. *P. aeruginosa* encodes a similar protein to BaeS (Locus: CRQ12647; 96% similarity), whose function remains unknown. Deletion of *baeSR* in *A. baumanii* led to a significantly reduced expression of the major efflux pumps, such as AdeABC, AdeIJK, and MacAB-TolC [218], resulting in increased susceptibility to tigecycline. The regulons of AdeRS and BaeSR overlap. This could also mean that BaeSR may function through crosstalk with AdeRS [217,218].

## 5. Two-Component Regulatory Systems as Potential Drug Targets

Two-component regulatory systems in gram-negative pathogens, though highly complex, may serve as attractive drug targets for a variety of reasons. The first reason is the high degree of structural and functional homology between various TCSs. A compound that is effective against a specific TCS, should plausibly be effective against other bacteria too, [271,272]. Secondly, TCSs regulate diverse, but essential functions in the cells and thus form a very effective target so that an inhibitor would inflict a global effect and not just targeting one pathway. Targeting a central regulatory system can significantly affect cell viability with low risk of development of quick resistance. Thirdly, many antimicrobial resistant genes are regulated either directly or indirectly by TCSs and targeting TCSs forms an excellent adjunct to currently available antimicrobials. Lastly, the possibility of negative side effects with the use of TCS targeting drugs are expected to be minimum as the bacterial histidine based TCS is very different from the eukaryotic serine/threonine based signaling systems. Thus, TCSs serve as an excellent target for drug development for combating microbial infections, including those resistant to currently available antibiotics.

Designing a drug-targeting specific TCSs is, however, complicated. The possible sites of intervention for a TCS are to be determined. Studies in the past have targeted the RR DNA binding [273], autophosphorylation sites [274] as well as ATP-binding domains [275]. Suggested sites for targeting include the site for autophosphorylation, site of interaction for HK-RR, facilitating the dephosphorylation of the HK, and inhibiting binding to the downstream genes. The question remains about the active sites for the known TCSs and whether they are common to all pathogens of one class. Targeting TCSs could be better if the targeted TCSs were conserved across several gram-positive and gram-negative bacterial species. Then there is the question about selectivity and spectrum of activity based on conservation of the active sites. Undoubtedly, further studies are needed before an antimicrobial drug targeting TCS is successfully developed.

## 6. Conclusions and Perspective

Studies of TCS signaling circuits continue to reveal new layers of complexity for these systems. The conventional notion of the TCSs is frequently being challenged by new findings and our understanding of bacterial signaling through TCSs and other related systems is continuously evolving. The complex connection of bacterial signaling systems and antibiotic resistance are expected to be revealed by further studies and so will the underlying mechanisms.

Autoregulation is an important concept in TCS signaling and has only recently begun to emerge and is well-illustrated by the BvgS system from the BvgS/BvgA system in *Bordetella bronchiseptica*. It was observed that autoregulation by BvgS modulates the sensitivity of the system to an applied stimulus [276]. Autoregulation can be positive or negative. Positive autoregulation occurs when a response regulator activates transcription of its own gene and the gene encoding its partner histidine kinase. In contrast, negative autoregulation is less common and involves a response regulator that represses its own expression. The other example of autoregulation within TCSs is the PhoQ/PhoP system in *E. coli*. In the steady state, output was unaffected by autoregulation over a wide range of (stimulus levels) magnesium concentrations. However, when faced with growth-limiting levels of magnesium, the autoregulation of histidine kinase, PhoQ amplified the output. When PhoQ was mutated to incapacitate phosphatase activity, there was strong amplification of autoregulation irrespective of the stimulus conditions [277]. Phosphorylation, though important, is not necessary for autoregulation. In the case of TorR [278] and LuxO [279], the response regulator can repress its own expression, irrespective of its phosphorylation status. Interestingly, autoregulation has also been said to result in a “short term memory” or a “learning behavior.” This means that once a signal has been perceived in the past, the bacteria form a memory of the stimulus and upon subsequent exposures respond faster or more extensively to a signal [280]. In the case where two proteins “cross-communicate” through the expression of an auxiliary protein, also known as two-component “connectors,” autoregulation may influence the system by controlling the relative concentrations of the interacting proteins. Understanding autoregulation is important because often it is mediated by highly conserved proteins and can be acquired by HGT. This is important to developing antimicrobial resistance amongst pathogens and must be considered when identifying viable drug targets.

Cross-talks of the TCSs and information exchange through direct interactions are other intriguing aspects of bacterial signaling mediated by TCSs and related systems. Is there a possibility of physiological cross-talk amongst TCSs in different species given their similarities in domains and functions? Further work is required in *P. aeruginosa*, *A. baumanii,* and *K. pneumoniae* to investigate the role of autoregulation, identify connectors in cross-talk of the TCSs, and its role in antimicrobial resistance. In this increasingly critical time of drug resistance, there is a growing need to understand the signals perceived by TCSs, their complex circuitries, as well as their modular architectures. New methods are to be developed to study TCS activity in vivo to answer questions about specificity and selectivity. In any case, TCSs promise a significant therapeutic target.

## Figures and Tables

**Table 1:** Key TCSs that are reportedly or potentially associated with virulence and/or antibiotics resistance in *P. aeruginosa*, *A. baumannii and K. pneumoniae*.

Name of the Two-Component System	Confirmed or Predicted Function	Reference(s)
***P. aeruginosa***		
PhoQ/PhoP	Regulating ABC transporter system; Resistance to antimicrobial peptides, polymyxins, and aminoglycosides; Regulating virulence, swarming motility and biofilm formation; Mg^2+^ sensing.	[130,131,132,133]
PmrA/PmrB	Activated by low Mg^2+^ and cationic antimicrobial peptides; Resistance against polymyxin B, colistin and other antimicrobial peptides	[131,134,135]
CpxA/CpxR	Role in cell envelope stress response; Activates MexAB-OprM efflux pump expression	[136]
CprS/CprR	Role in LPS modification and antimicrobial peptide resistance	[137]
ParR/ParS	Role in resistance to colistin and polymyxins; Role in quorum sensing, phenazine production, and motility	[137,138]
GacS/GacA	Regulating virulence factors; Biofilm formation; Antibiotic resistance; Motility; Iron metabolism; Type III and type VI secretion	[139]
PvrS/PvrR	Regulation of the MexAB-OprM efflux pump; Biofilm formation. Controls of fimbrial genes	[140,141,142,143]
RcsC-RcsB	Role in biofilm formation and control of fimbrial genes	[140,141]
AmgS-AmgR	Involved in aminoglycoside resistance and cell envelope stress response	[144,145,146]
PA1611	Biofilm formation and virulence regulation	[96,129]
BfiS/BfiR	Biofilm maturation	[147]
HptB/HsbR	Involved in swarming motility and biofilm formation	[148,149]
RocS2/RocA2	Regulation of fimbrial adhesins and antimicrobial resistance	[143,150]
ErbR/EraR	Control of biofilm specific antibiotic resistance	[151]
TctE/TctD	Controls expression of tricarboxylic acid (TCA) uptake system	[86]
PhoR–PhoB	Plays a role in quorum sensing and swarming motility	[86,152]
ChpA/PilG/PilH/ChpB	Regulation of the chemosensory pili (Pil–Chp) system, twitching motility and cAMP levels; Regulates virulence genes	[153,154]
FimS (AlgZ)/AlgR	Regulation of virulence; Alginate biosynthesis; Motility; Biofilm formation; Cytotoxicity and type III secretion system expression	[155]
ColS/ColR	Polymyxin resistance; Virulence and cell adherence	[20]
CreC–CreB	Role in catabolism; Swarming and swimming motility; Antibiotic resistance; Biofilm and global gene regulation	[156]
PirR–PirS	Iron acquisition	[157]
FleS–FleR	Flagellar motility; Adhesion to mucins	[158]
PA1396/PA1397	Plays a role in interspecies signaling; Responds to diffusible signal factor (DSF); Regulates biofilm formation and antibiotic resistance	[159]
CzcS–CzcR	Regulates heavy metal resistance; Controls antibiotic resistance and pathogenicity	[160,161]
RetS	Regulates virulence; Biofilm formation; Regulates Type III and VI secretion/cytotoxicity	[99,162]
LadS	Regulates virulence; Biofilm formation; Type III secretion/cytotoxicity	[99,101]
BqsS/BqrR/CarS/CarR	Biofilm formation; Iron sensing; Antibiotic resistance and cationic stress tolerance. Maintains Ca^2+^ homeostasis; Regulates pyocyanin secretion; Motility.	[163]
PfeS–PfeR	Iron acquisition	[87]
CopS–CopR	Tolerance to Cu^2+^, Zn^2+^; Imipenem resistance	[161]
GtrS/GltR	Regulates glucose transport and Type III secretion system	[164,165]
WspE–WspR	Regulates biofilm formation, autoaggregation, and cyclic-di-GMP synthesis	[166,167,168]
NarX–NarL	Nitrate sensing and respiration; Biofilm formation; Motility	[84]
BfmS/BfmR	Biofilm formation/maintenance	[147]
PprA–PprB	Regulates outer membrane permeability; Aminoglycoside resistance; Controls virulence including type III secretion system and biofilm formation	[169,170,171]
RoxS/RoxR	Confers cyanide tolerance	[172]
PilS–PilR	Involved in regulating the expression of the T4P major subunit PilA; Biofilm formation; Motility; Positively regulates the transcription of flagellar regulatory genes	[173]
CbrA–CbrB	Metabolic regulation of carbon and nitrogen utilization. Modulates biofilm formation; Cytotoxicity; Motility; Antibiotic resistance	[89]
AruS/AruR	Controls the expression of the arginine transaminase pathway	[174,175]
NtrB/NtrC	Responds to cellular nitrogen levels and activates nitrogen scavenging genes	[176]
DctB/DctD	Controls the expression of C4-dicarboxylate transporters	[177]
KinA/AlgB	Regulates alginate biosynthesis; Regulates virulence	[178]
MifS/MifR	Role in biofilm formation and metabolism	[179]
***K. pneumoniae***		
CpxA/CpxR	Sensing extracellular pH and membrane composition; Regulating cell envelope protein folding and protein degradation	[180,181]
PhoP/PhoQ	Activates *pmrHFIJKLM;* Responsible for L-amino arabinose synthesis and polymyxin resistance	[182,183,184]
PhoR/PhoB	Phosphate assimilation	[180]
QseC/QseB	Involved in regulation of the flagella and motility genes	[185]
KvgA/KvgS	Involved in tolerating free radical stresses and sensing iron-limiting conditions	[186]
KvhA/KvhS	Regulates capsular polysaccharide synthesis	[187,188]
PmrA/PmrB	Regulator of genes for lipopolysaccharide modification	[189]
RcsC/RcsB	Involved in the capsular polysaccharide biosynthesis; Type III system; Regulates the production of major pilin protein MrkA; Confers resistance to low pH	[190]
EnvZ/OmpR	Senses osmotic signals; Regulates the c-di-GMP signaling pathway; Regulates type III fimbriae and biofilm formation	[191,192,193]
CusS/CusR	Induced by Copper and regulates the CusCFBARS efflux system; Tolerance to silver	[194,195,196]
KdpD/KdpE	Potassium transporter system	[197,198,199]
BaeS/BaeR	Regulates Multidrug efflux pump AdeABC; Regulates Modification of lipopolysaccharides	[199,200]
ArcB/ArcA	Involved in modulating the expression of genes encoding for proteins with membrane modification functions and TCA cycle enzymes depending upon oxygen levels.	[199,201]
NarX/NarL	Role in nitrate and nitrite reductase synthesis	[202,203]
UhpB/UhpA	Role in uptake of hexose phosphates	[199,204,205]
EvgS/EvgA	Regulates capsular polysaccharide biosynthesis	[206,207]
GlnL/GlnG	Role in glutamate metabolism	[208,209]
ZraR/ZraS	Zinc-responsive TCS; Activated under high calcium and iron conditions	[210]
CitA/CitB	Regulates citrate metabolism under anaerobic conditions	[211,212]
CrrA/CrrB	Involved in polymyxin resistance	[213]
***A. baumannii***		
PmrA/PmrB	Regulates genes involved in lipopolysaccharide modification	[214,215]
AdeS/AdeR	Regulates genes encoding the AdeABC pump	[59,216]
BaeS/BaeR	Stress response under high osmotic conditions	[60,217,218]
BfmS/BfmR	Regulates biofilm formation and antibiotic resistance	[219,220,221]
GacS/GacA	Regulates genes associated with pili and biofilm development, motility and resistance against host antimicrobial peptides	[222,223]
A1S_2811	Involved in surface motility and biofilm formation	[224]
KdpD/KpdE	Regulates potassium transport	[225]
GlnL/GlnG	Involved in nitrogen assimilation	[226]
PhoR/PhoB	Regulates phosphate assimilation	[227]
CusS/CusR	Senses copper ions and upregulates the expression of an RND family efflux pump that removes copper ions from the cell	[228]
OmpR/EnvZ	Regulates virulence; Phase variation; Osmotic tolerance	[229]

## Abbreviations

AAC	Aminoglycoside Acetyltransferase
ABC	ATP Binding Cassette
AMR	Antimicrobial Resistance
CAPs	Cationic Antimicrobial Peptides
CDC	Centers for Disease Control and Prevention
CF	Cystic Fibrosis
CHDL	Carbapenem-Hydrolysing Class D β-Lactamase
Csr	Carbon Storage Regulation
DSF	Diffusible Signal Factor
ESBL	Extended-Spectrum β-lactamase
ESCAPE	*Enterococcus faecium*, *Staphylococcus aureus*, *Clostridium difficile*, *A. baumannii, P. aeruginosa*, and *Enterobacteriaceae*
ESKAPE	*Enterococcus faecium*, *Staphylococcus aureus*, *K. pneumoniae*, *A. baumannii, P. aeruginosa,* and *Enterobacteriaceae*
HAP	Histidyl-Aspartyl Phosphorelay
HGT	Horizontal Gene Transfer
HHK	Hybrid Histidine Kinase
HK	Histidine Kinase
Hpt	Histidine Phosphotransfer Protein
HTH	Helix-turn-helix
ICU	Intensive Care Unit
L-Ara4N	4-Amino-4-Deoxy-L-Arabinose
LPSs	Lipopolysaccharides
MATE	Multidrug and Toxin Extrusion
MBL	Metallo β-lactamases
MDR	Multidrug-Resistant
MFS	Major Facilitator Superfamily
NDM-1	New Delhi Metallo- β-lactamase 1
NNIS	National Nosocomial Infections Surveillance
OCSs	One-Component Systems
OM	Outer Membrane
PAA	Phenylacetic Acid
PAS	Period clock protein, Aryl hydrocarbon receptor, and Single-minded protein
PDR	Pan-Drug Resistant
pEtN	Phosphoethanolamine
RND	Resistance-Nodulation-Cell Division
RR	Response Regulator
SCV	Small Colony Variants
SMR	Small Multidrug Resistance
TCA	Tricarboxylic Acid
TCSs	Two-Component Systems
WHO	World Health Organization
